# Comparing Living Experiences in Subdivided Unit and Public Housing in Hong Kong: A Qualitative Study

**DOI:** 10.1093/geroni/igaf122.3904

**Published:** 2025-12-31

**Authors:** Peiyi Lu

**Affiliations:** University of Hong Kong, Hong Kong, Hong Kong, China (People’s Republic)

## Abstract

Crowded housing conditions have been linked to poorer health, with these effects potentially intensifying in older age. Yet, existing research has largely focused on general adult population without comparing aging experiences across housing types. This study explores how housing environments shape aging by comparing middle-aged and older adults (aged 45+) living in subdivided units (SDUs) and public housing in Hong Kong. Thirty participants—15 from SDUs and 15 from public housing—were purposefully selected. Face-to-face interviews using open-ended questions explored how individuals perceived their housing conditions to affect their daily lives, health, and social relationships. Thematic analysis was conducted to identify key patterns. Most participants had low incomes and received government aid. SDU residents, mainly single men in bedspace apartments, reported strained family ties and poorer living conditions, including noise, unsanitary environments, air pollution, and lack of elevators. As they aged, they expressed a need for age-friendly housing with more space, cleanliness, privacy, and accessibility. In contrast, public housing residents were generally satisfied with their living environments. The findings suggest that public housing offers a more supportive environment for aging than SDUs. The inadequate conditions in SDUs pose risks to physical and mental health as individuals grow older. To promote healthy aging, it is recommended that the government strengthen and enforce building regulations for subdivided units to meet aging-related health standards.

